# Cutaneous infection by *Mycobacterium lentiflavum* after subcutaneous injection of lipolytic formula^☆^^☆☆^

**DOI:** 10.1016/j.abd.2019.11.013

**Published:** 2020-05-13

**Authors:** Renan Bernardes de Mello, Dalton Nogueira Moreira, Ana Carolina Gomes Pereira, Nicole Ramalho Lustosa

**Affiliations:** a Postgraduate Program in Health Sciences, Faculdade de Medicina, Hospital das Clínicas, Universidade Federal de Minas Gerais, Belo Horizonte, MG, Brazil; b Orestes Diniz Training and Reference Center for Infectious and Parasitic Diseases, Hospital das Clínicas, Universidade Federal de Minas Gerais, Belo Horizonte, MG, Brazil; c Department of Clinical Medicine, Hospital Semper, Belo Horizonte, MG, Brazil; d Dermatology Service, Hospital da Polícia Militar de Belo Horizonte, Belo Horizonte, MG, Brazil

**Keywords:** Mesotherapy, Mycobacterium infections, nontuberculous, Soft tissue infections

## Abstract

The incidence of nontuberculous mycobacterial infections is increasing worldwide; by 2017, more than 190 species and subspecies have been documented. Although classically associated with immunosuppression, the recognition of these etiological agents in diseases affecting immunocompetent individuals and in healthcare-associated infections, such as after surgical and cosmetic procedures, makes the study of the epidemiology and pathogenesis of these microorganisms relevant in medical practice. *Mycobacterium lentiflavum* is slow-growing and rarely affects the skin. A case of cutaneous mycobacteriosis caused by *M. lentiflavum* is reported in an immunocompetent patient after subcutaneous injection of a lipolytic compound, treated with clarithromycin and levofloxacin.

Atypical mycobacterioses are caused by heterogeneous species of mycobacteria that can be classified through various criteria, such as slow-growing nontuberculous mycobacteria and fast-growing mycobacteria, pigment production, colony morphology, and other biochemical tests.^1^, ^2^ In addition to nontuberculous mycobacteria (NTM), the obligate pathogens of the *Mycobacterium tuberculosis* complex and *Mycobacterium leprae* also belong to the genus *Mycobacterium*, causing tuberculosis and leprosy, respectively.^1^, ^2^ With the application of molecular biology techniques, among them high-performance liquid chromatography (HPLC) of mycolic acids, polymerase chain reaction (PCR), restriction enzyme, and genetic sequencing, new species of NTM have been described, broadening the understanding of its ecology, microbiology, and significance in medical practice.^3^ In the clinical setting, species recognition is relevant due to intrinsic resistance of NTM to current anti-tuberculosis regimens.^2^

NTMs are generally free-living saprophytes and have been isolated in water, soil, aerosols, and on objects, including medical utensils and equipment.^4^, ^5^ NMT infections have already been reported as complications in the following procedures: cardiac and ophthalmologic surgery, liposculpture/liposuction, mammoplasty, tattooing, application of botulinum toxin and fractionated CO_2_ laser, skin filling, mesotherapy, skin biopsy, Mohs surgery, pedicure, acupuncture, piercing implant, and variceal sclerotherapy.^4^, ^5^, ^6^

In this context, fast-growing species are often isolated, such as the *M. fortuitum*, *M. abscessus*, and *M. chelonae*.^4^, ^5^ Unlike the fast-growing NTMs, *M. lentiflavum* is a slow-growing bacteria, which has been associated with superficial lymphadenitis in children and pulmonary infections, mainly in immunosuppressed patients.^7^, ^8^, ^9^ However, cutaneous mycobacteriosis due to *M. lentiflavum* is rare and was first reported in an HIV-infected patient with a CD4+ T lymphocyte count of 46/mm^3^, by Montejo et al. in 2006.^10^

The present report details the case of a healthy 28-year-old woman who underwent subcutaneous applications of a substance composed of 5% sunflower oil, 6% deoxycholate, 5% sinetrol, and caffeine 50 mg in the abdomen and flanks by a non-medical professional, with weekly intervals between sessions. After the fourth application, she presented pain, erythema, and heat at the infiltration site (Fig. 1A and B). There was further worsening of pain and edema, associated with drainage of a purulent secretion (Fig. 2A and B).

**Figure 1:**
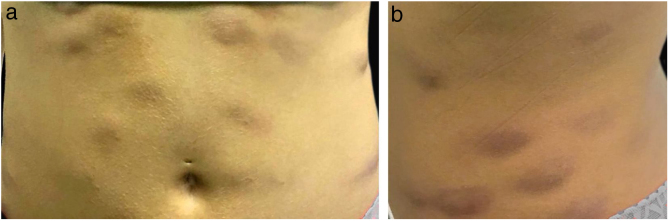
(A and B) Erythematous and infiltrated nodules in the abdomen.

**Figure 2:**
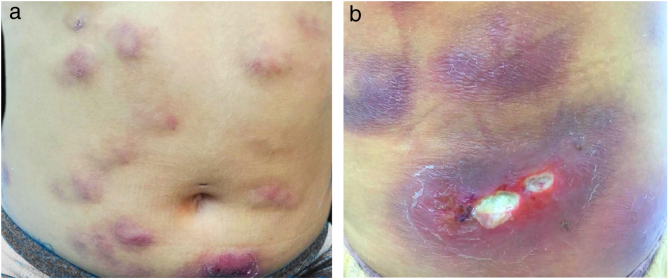
(A and B) Erythematous and infiltrated nodules and plaques, some with ulceration and suppuration.

The direct microscopic examination and culture for fungi and bacteria of the lesion secretion in the abdomen, besides the serologies for viral hepatitis and HIV, were negative. The acid-fast bacilli smear (AFB) was positive in two different samples. The PCR test for *M. tuberculosis* was negative and the chest X-ray showed no abnormalities. Then, empiric treatment for atypical mycobacteriosis was decided upon, using clarithromycin 500 mg twice daily associated with levofloxacin 500 mg once daily, as well as debridement of the lesions. The histopathological study revealed chronic granulomatous and suppurative inflammation, with organized abscesses, absence of vasculitis, and negative findings for specific microorganisms (Fig. 3A and B). The restriction fragmentation length polymorphism (RFLP) analysis of DNA generated by PCR was compatible with *Mycobacterium lentiflavum*. After two months of treatment, there was partial improvement of the condition (Fig. 4A), and at the end of an eight-month course of treatment, complete remission with atrophic scars was observed (Fig. 4B).

**Figure 3:**
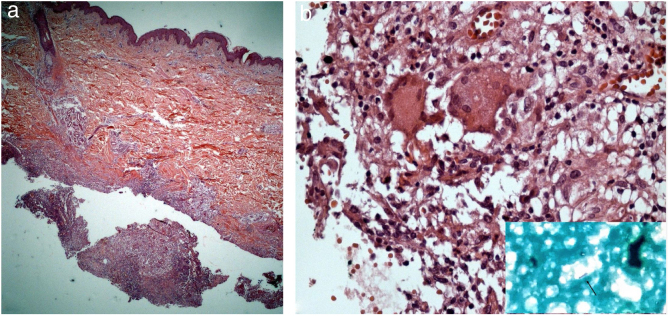
(A) Superficial and deep lymphohistiocytic and granulomatous infiltrate, with suppuration and organized abscesses (Hematoxylin & eosin, x200). (B) In detail, acid-fast bacilli are noted in the purulent secretion (Hematoxylin & eosin, x400).

**Figure 4:**
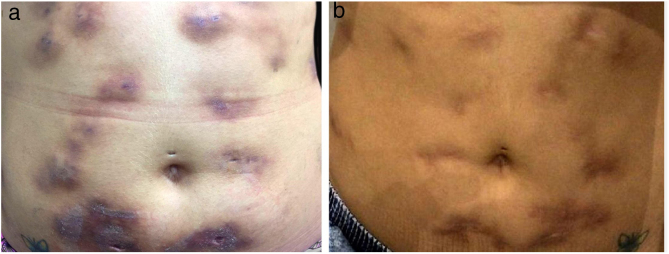
(A) Decreased inflammatory process after 2 months of antibiotic therapy. (B) Disseminated atrophic scars at the end of an 8-month course of treatment.

## Financial support

None declared.

## Authors' contributions

Renan Bernardes de Mello: Approval of final version of the manuscript; conception and planning of the study; drafting and editing of the manuscript; critical review of the literature; critical review of the manuscript.

Dalton Nogueira Moreira: Approval of final version of the manuscript; conception and planning of the study; drafting and editing of the manuscript; intellectual participation in the propaedeutic and/or therapeutic conduct of the studied cases; critical review of the manuscript.

Ana Carolina Gomes Pereira: Approval of final version of the manuscript; conception and planning of the study; drafting and editing of the manuscriptcritical review of the manuscript.

Nicole Ramalho Lustosa: Approval of final version of the manuscript; conception and planning of the study; drafting and editing of the manuscriptcritical review of the manuscript.

## Conflicts of interest

None declared.

## References

[1] Bhambri S., Bhambri A., Del Rosso J.Q. (2009). Atypical mycobacterial cutaneous infections. Dermatol Clin.

[2] Riello F.N. (2015). Identificação molecular de espécies de micobactérias por PCR-RFLP hsp65 e implicações clínicas do diagnóstico convencional.

[3] Kothavade R.J., Dhurat R.S., Mishra S.N., Kothavade U.R. (2013). Clinical and laboratory aspects of the diagnosis and management of cutaneous and subcutaneous infections caused by rapidly growing mycobacteria. Eur J Clin Microbiol Infect Dis.

[4] Gonzalez-Santiago T., Drage L.A. (2015). Nontuberculous mycobacteria: skin and soft tissue infections. Dermatol Clin.

[5] Cabral D., Andrade D. (2011). Nontuberculous mycobacteria in surgery: challenges likely to be faced in Brazil?. Acta Paul Enferm.

[6] Murback N., Higa Júnior M., Pompílio M., Cury E., Hans Filho G., Takita L. (2015). Disseminated cutaneous atypical mycobacteriosis by *M. chelonae* after sclerotherapy of varicose veins in a immunocompetent patient: a case report. An Bras Dermatol.

[7] Molteni C., Gazzola L., Cesari M., Lombardi A., Salerno F., Tortoli E. (2005). *Mycobacterium lentiflavum* infection in immunocompetent patient. Emerg Infect Dis.

[8] Yagi K., Morimoto K., Ishii M., Namkoong H., Okamori S., Asakura T. (2018). Clinical characteristics of pulmonary *Mycobacterium lentiflavum* disease in adult patients. Int J Infect Dis.

[9] Tortoli E., Mattei R., Russo C., Scarparo C. (2006). *Mycobacterium lentiflavum*, an emerging pathogen?. J Infect.

[10] Montejo M., Goicoetxea J., Agesta N., Gil A., Urra E., Jimenez M.S. (2006). Cutaneous infection by *Mycobacterium lentiflavum* in a patient with HIV. Dermatology.

